# Preparation and Antibacterial Properties of a Composite Fiber Membrane Material Loaded with Cationic Antibacterial Agent by Electrospinning

**DOI:** 10.3390/nano13030583

**Published:** 2023-02-01

**Authors:** Lin Li, Chengfu Zhang, Lina Tian, Zihang Wu, Dongqing Wang, Tifeng Jiao

**Affiliations:** State Key Laboratory of Metastable Materials Science and Technology, Hebei Key Laboratory of Applied Chemistry, Hebei Key Laboratory of Nano-Biotechnology, Hebei Key Laboratory of Heavy Metal Deep-Remediation in Water and Resource Reuse, Yanshan University, Qinhuangdao 066004, China

**Keywords:** electrospinning, composite nanofibers, cationic antibacterial agent, antibacterial

## Abstract

Microbial infections due to bacteria, viruses, and molds are a serious threat to both human life and the health of other organisms. To develop inexpensive, easy-to-prepare, efficient, and portable nano-antibacterial materials, as well as to explore the antibacterial prospects of cationic antibacterial agents, in this work, six different membrane materials were prepared by the electrostatic spinning method and characterized by scanning electron microscopy (SEM), transmission electron microscopy (TEM), X-ray diffraction (XRD), and Fourier transform infrared (FT-IR). The materials were tested for antimicrobial properties using a modified AATCC100-200 test method. Under the most suitable spinning conditions, the doping amount of the cationic antimicrobial agent, CTAB, had the greatest influence on the antimicrobial performance. The antimicrobial performance of PCL/PEO/CS/CTAB_0.4_ was the highest among the prepared materials, with 83.7% effectiveness against *S. aureus* and 99.9% against *E. coli*. The antimicrobial performance was found to be stable. In our study, we determined the most suitable spinning ratio to prepare an inexpensive and efficient cationic antimicrobial agent. Biodegradable, high-antimicrobial-activity antimicrobial materials can be applied as films, and this new nanofiber material has shown great potential in wound dressings and as a mask material due to its remarkable antimicrobial efficiency.

## 1. Introduction

Bacteria are ubiquitous in our daily lives, and bacterial infections are one of the major contributing factors to human deaths worldwide [1]. The major cause of microbial infections is the widespread presence of microorganisms surrounding us. Once inside the body, these microorganisms develop and rapidly form colonies. They can easily enter the body through open wounds and infiltrate deeper tissue to cause internal infections [2]. The main challenge in wound care is preventing infection, which can lead to exudate formation, delayed wound healing, or disfigurement, or even be life-threatening [3]. According to the World Health Organization (WTO), bacterial infections have been one of the main causes of disease and death in less developed countries and regions in recent years [4]; therefore, the development of materials with antimicrobial properties is essential. The ideal antimicrobial material should provide a moist environment to enhance the therapy [5] and have broad-spectrum antimicrobial activity [6], including activity against antibiotic-resistant bacteria [7]. Developments in nanoscience and nanotechnology can improve the materials and designs used in topical wound care, to effectively release antimicrobial [8], anti-inflammatory, and regenerative compounds to accelerate the endogenous healing process. Electrospinning is one of the main techniques for preparing nanoscale materials that is not only efficient and simple, but also easy to set up and operate [9,10]. 

Electrostatic spinning is an effective technique used to produce sequential nanofibers of 5–100 nm in length. Electrostatic spinning and related techniques for polymer fibers have recently been used to obtain new materials that have potential applications in medicine [11], energy [12,13], and environmental issues [14]. The most attractive features of these materials are their morphology-related characteristics, such as a fiber diameter in the submicron range [15], high porosity voids with interconnections [16], and large surface area/volume ratios [17]. A greater surface area and shorter diffusion channel length can improve the overall release rate of nanofiber drug systems compared to most conventional materials [18]. These features allow the material to better kill harmful bacteria and improve the efficiency of material utilization. Therefore, electrostatic spinning technology has been heavily invested in during the development of antimicrobial materials. 

Silver nanoparticles and other precious metal particles have good antimicrobial activity [19] and are often prepared as antimicrobial components in antimicrobial materials [20], but their scarcity and high prices limit their application [21,22]. Natural antimicrobial agents have poor antimicrobial performance and bacterial resistance. Therefore, it is imperative to find alternatives to antimicrobial agents with excellent performance, low cost, and less susceptibility to bacterial resistance. Cationic antimicrobial polymers are important functional polymers that are widely used in wastewater treatment, medical devices, and fiber textiles [23,24]. 

Cationic antimicrobial polymers can kill/inhibit the growth of microorganisms on their surface or in the surrounding environment, and they can be incorporated into composite spinning films to form inexpensive, efficient, portable, and simple antimicrobial materials. Chitosan (CS) is a natural cationic antibacterial polymer with good solubility [25], reactivity [26], and antibacterial activity [27] against various common bacteria [28,29], but its antibacterial performance is poor [30]. Therefore, cetyl trimethyl ammonium bromide (CTAB) was chosen to enhance the antibacterial performance of the spun fiber as a cationic surfactant. Cetyl trimethyl ammonium bromide (CTAB) is chemically stable [31], heat resistant [32], light-resistant, pressure resistant, resistant to strong acids and bases [33], easily soluble in isopropyl alcohol [34], soluble in water [35], has excellent permeability [36], and has softening, emulsification, antistatic, biodegradability, and bactericidal properties [37]. A cationic surfactant can adsorb anionic bacteria [38,39], destroy the cell membrane of the bacteria, and eventually lead to the autolysis and death of the bacteria [40], as well as causing the proteins of the bacteria to denature and precipitate [41]. 

In this study, PCL/PEO-based polymers were doped with CS and CTAB to enhance their solubility and antimicrobial activity. This material was not only antibacterial, but also optimized the microscopic morphology of the spun fibers. In addition, it also had good chemical stability, good solubility to various solvents, good permeability, biodegradability, and bactericidal properties.

## 2. Materials and Methods

### 2.1. Materials

Polycaprolactone (PCL, average Mw-80000), polyethylene oxide (PEO, average Mw-200,000), formic acid (analytical grade, purity > 98%), chitosan (CS, 95% deacetylated), and cetyl trimethyl ammonium bromide (CTAB, purity > 99%) were purchased from Shanghai Aladdin Bio-Chem Technology Co., Ltd. (Shanghai, China).

### 2.2. Preparation of Composite Spinning Fibers

A total of 0.5 g of PCL particles and 0.2 g of PEO powder were added to the formic acid solution and stirred at a constant temperature of 60 °C with a temperature-controlled magnetic stirrer. When the solution changed from turbid to colorless, clear, and transparent, the PCL/ PEO spinning precursor solution configuration was complete. A total of 0.1 g of CS powder was added to 4 mL of the formic acid solution and stirred until the solution turned light yellow; then, the mixed solution was added to the previously prepared PCL/PEO solution for 10 h, giving a PCL/PEO/CS solution. A total of 0.2 g of CTAB powder was added to PCL/PEO solution until the solution was clear, indicating that the PCL/PEO/CTAB_1_ solution had been prepared. A total of 0.2 g, 0.4 g, and 0.6 g of CTAB powder was added to three identical 10 mL PCL/PEO/CS solutions and stirred until the solution was clarified at the normal temperature. The three spinning precursor solutions of PCL/PEO/CS/CTAB_0.2_, PTCL/PEO/CS/CTAB_0.4,_ and PCL/PEO/CS/CTAB_0.6_ were thus prepared. The above six spinning materials were prepared using 18 gauge syringes with a spinning volume of 2 mL, with a spinning speed of 0.4 mL/h for PCL/PEO and PCL/PEO/CS; a spinning speed of 0.5 mL/h for PCL/PEO/CTAB_1_ and PCL/PEO/CS/CTAB_0.2_; a spinning speed of 0.6 mL/h for PCL/PEO/CS/CTAB_0.4_ and PCL/PEO/CS/CTAB_0.6_; a receiving distance of 15 cm for PCL/PEO, PCL/PEO/CS, and PCL/PEO/CTAB_1_; and a receiving a receiving distance of 16 cm for PCL/PEO/CS/CTAB_0.2_, PCL/PEO/CS/CTAB_0.4_, and PCL/PEO/CS/CTAB_0.6_.

Finally, the prepared electrospun films were dried at room temperature and subsequently tested for characterization and antibacterial properties.

### 2.3. Characterizations

The morphology of the composite films was measured by scanning electron microscope (SEM-S-4800II, JOEL, Tokyo, Japan) at an accelerating voltage of 15 kV. The surfaces of the composite films were coated with platinum (Pt) in a sputtering chamber for 120 s at 30 mA. In addition, elemental analysis was studied using energy dispersive X-ray spectroscopy (EDS). During the spinning process, the copper mesh was fixed with forceps to receive the spun sample, and then the spinning sample morphology was further observed with a transmission electron microscope (TEM). We cut the appropriately sized samples and lay them flat on the stage to analyze the crystal structure with an X-ray diffractometer (XRD, Smart Lab, Nippon Rigaku Corporation, Tokyo, Japan) operating at 30 kV and 500 mA, at a 2ff ranging from 5° to 80°. Fourier infrared spectroscopy (FTIR, Nicolet iS10, Thermo scientific, Waltham, MA, USA) in the range of 4000 to 400 cm^−1^, with accumulation over 20 scans, was used to analyze the samples via infrared spectroscopy to further verify the structure of the substance. We analyzed the elements contained in the sample by Mapping. The water contact angle was tested with an optical contact angle measuring instrument to determine the hydrophobic properties of the sample.

### 2.4. Antibacterial Assays

The culture dishes, tweezers, and other tools used in the antibacterial performance test experiment were wrapped in aluminum foil, and the configured nutrient broth, physiological saline, and other solutions were sealed with sealing films. They were placed together in a vertical-pressure steam sterilizer for sterilization. The sterilization temperature was 121 °C, the sterilization time was 15 min, and the sterilized tools and utensils were placed in a vertical blast drying oven at 50 °C on dry standby. The sterilized solution was cooled on an ultra-clean bench for later use. The bacteria used in the experiment were *Escherichia coli* and *Staphylococcus aureus*. All operations in the experiment were carried out on an ultra-clean bench.

After sterilization of all equipment, the antibacterial properties of the composite films were analyzed by bacteriophage test, and the experimental operation was divided into the following steps. The bacterial seed solution was first prepared and refrigerated. The refrigerated bacterial seed solution was then placed in the nutrient broth until the OD_600_ value of the bacterial solution was between 0.4 and 0.5. Finally, the antibacterial effect of the six electrospinning membranes was judged by observing the antibacterial sphere. After the qualitative analysis of the antibacterial properties of the composite films, the antibacterial activity was tested again. The antimicrobial rates of the six spinning membranes were tested using *S. aureus* and *E. coli* to assess the antibacterial activity of the spinning fibers synthesized from the different components. Finally, the bacterial growth curves of the six spinning membranes and the bacterial control group were drawn over 48 h to observe each sample’s influence on the bacterial growth curve.

*S. aureus* and *E. coli* were used to test the bactericidal efficacy of the six spinning membranes, and the inoculation amount of each sample was about 106 CFU bacteria. The modified AATCC100-2004 test method was utilized [42]. First, each of the six spinning membranes was cut into a 2.25 cm^2^ square, then laid flat in a sterilized Petri dish and sterilized under an ultraviolet lamp for 30 min. Then, a bacterial solution with an OD_600_ value of 0.4–0.5 was configured, and 25 uL of bacterial droplets was removed by a pipette gun and spread in the center of the spinning membrane. After 10 s of contact, the sample was placed in a test tube containing 40 mL of normal saline, the lid was tightened, and a rotating vortex was used to remove the residual inoculum from the spinning membrane into the solution. A 10 times continuous dilution was performed using normal saline. We then took 1 mL of each diluent to a Petri dish and added 20 mL to 50 °C nutrient agar. We then placed the Petri dish on the clean bench and gently shook it to mix the broth with nutrient agar. Three copies were made, and 10 min after agar coagulation, we placed the samples upside down in a 37 °C constant temperature incubator. They were removed 24 h later and the appropriate dilution factor was selected (the number of bacteria was between 30–300) to observe the number of bacteria, calculate the average number of bacterial colonies to analyze the antibacterial effect, and calculate the antibacterial rate of the six kinds of spinning membranes. 

For the positive control experiment, we used a pipette to remove 25 uL of bacteria with an OD_600_ value between 0.4 and 0.5, and we added this to 40 mL saline to absorb the solution for a continuous gradient dilution. Three copies were made to find the appropriate gradient count to determine the actual number of bacteria. For the negative experiment, we used normal saline to repeat the same experimental steps, and 24 h after taking it out from the incubator, observed the culture dish to ensure no bacteria had grown.

We configured six bottles of 50 mL nutrient broth, each with 1 mL of bacterial seed solution added, along with six types of sterilized 2.25 cm^2^ square spinning membrane samples added with tweezers. In another bottle of nutrients, 1 mL of bacterial seed liquid only was added as the control group. The seven bottles of nutrient broth were sealed with sealing films and shaken in a 37 °C,160 r/min gas bath thermostatic oscillator. In the first 12 h, the OD595 value of the bacterial solution was measured every 2 h (200 uL of the bacterial solution was placed in a 96-well plate with a pipette and detected by a microplate reader), and the determination was repeated three times. After 12 h, the time interval was changed to 12 h, and the test lasted 48 h in total. The bacterial growth curve of the six kinds of spinning films and the bacterial control group over 48 h was drawn, with the measurement time as the abscissa and the absorbance as the ordinate, and the influence of the sample on the bacterial growth curve was observed.

## 3. Results and Discussion

### 3.1. Structural Characterization of Composite Spinning Films 

**Figure 1:**
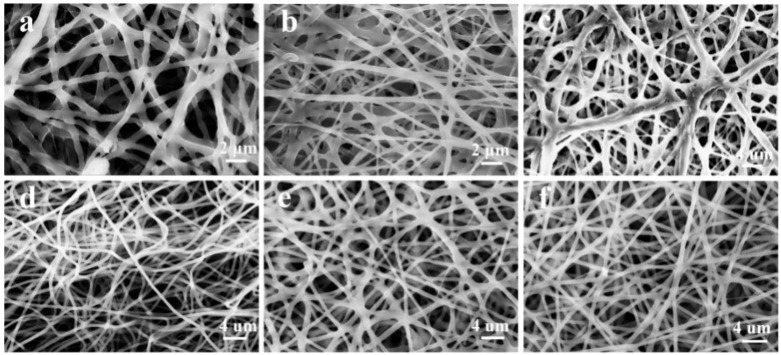
SEM images of composite spinning films: (**a**), PCL/PEO; (**b**), PCL/PEO/CS; (**c**), PCL/PEO/CTAB_1_; (**d**), PCL/PEO/CS/CTAB_0.2_; (**e**), PCL/PEO/CS/CTAB_0.4_; (**f**), PCL/PEO/CS/CTAB_0.6_.

We then performed a series of structural characterizations of the six prepared composite spinning films. Figure 1a shows SEM images of the PCL/PEO composite spinning films. It can be seen from the images that the PCL/PEO composite films had a non-uniform diameter, random orientation, and uneven fiber surface, leading to light and dark non-uniformity in the scanned images. From Figure 1b, the fibers of PCL/PEO/CS electrostatic spinning film were flatter than those of PCL/PCL, with fewer bright and dark parts in the image and a smooth composite spinning film surface, but the diameter was different, and the thickness was not uniform. The composite spinning film diameters of PCL/PEO/CTAB shown in Figure 1c were also uneven, but the average diameter was larger than that of the first two composite spinning films. Figure 1d,e were all spun from the same component, and only the amount of CTAB was changed. From the figures, it can be seen that the PCL/PEO/CS/CTAB composite spinning film had a more regular microscopic morphology than the two-component and three-component composite spinning films. With the increase of CTAB dosage, the composite spinning film arrangement became more orderly, the fiber surface was smooth, the surface of the composite spinning film membrane was flatter, and the composite spinning film thickness was increasingly uniform. In addition, the high porosity and high specific surface area of the composite spinning film also helped to contact bacteria and enhanced the sterilization rate.

**Figure 2:**
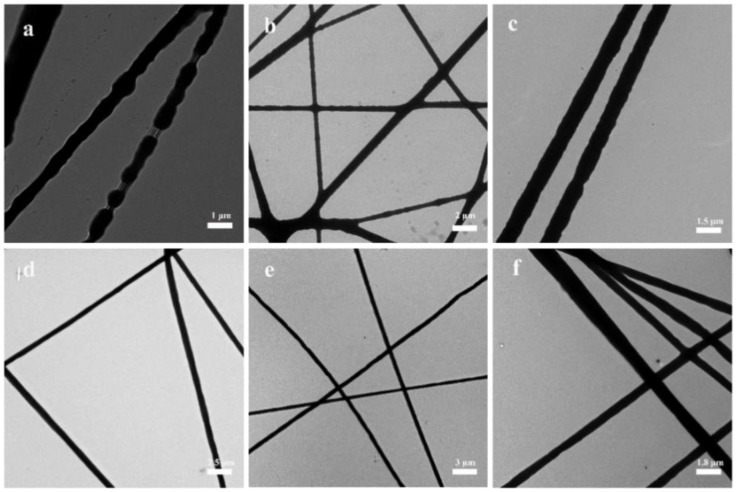
TEM images of the composite spinning films: (**a**), PCL/PEO; (**b**), PCL/PEO/CS; (**c**), PCL/PEO/CTAB_1_; (**d**), PCL/PEO/CS/CTAB_0.2_; (**e**), PCL/PEO/CS/CTAB_0.4_; (**f**), PCL/PEO/CS/CTAB_0.6_.

To observe the structure of the composite spinning film more clearly, we performed another TEM test. From Figure 2a, it can be observed that the PCL/PEO composite spinning films showed a broken point situation, and the spun filaments were not coherent, with diameters between 0.7 um and 1.5 um. The composite spinning films in Figure 2b had a smooth surface and a non-uniform diameter size between 0.5 um and 1.0 um. Figure 2c shows that the composite spinning films matched the SEM characterization results, with smooth fiber surfaces and diameters around 1.0 um. The electrostatic spinning shown in Figure 2d–f corresponds to the scanned images in Figure 1d–f. With the increase of CTAB, the composite spinning films showed a more orderly arrangement, the surface was smoother and flatter, and the composite spinning films were more uniform in thickness, with diameters of 0.8, 0.5, and 0.9 um, respectively. They had a high specific surface area, which helped them contact bacteria and enhanced the sterilization rate.

**Figure 3:**
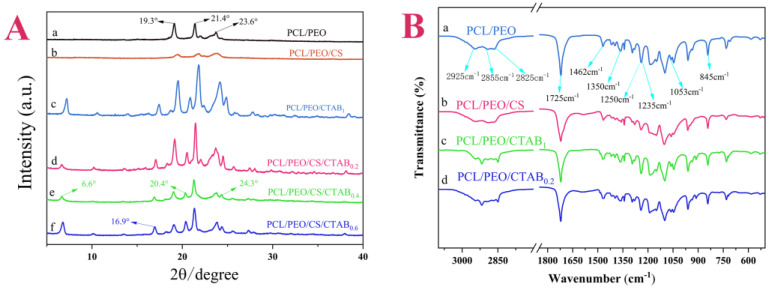
(**A**), XRD patterns of composite spinning films; (**B**), FT-IR spectra of composite spinning films.

To determine whether the composite spun films were prepared successfully, XRD characterization tests were performed, and the results obtained are shown in Figure 3A. The XRD characteristic diffraction peaks of PCL were found to be at 21.4° and 23.7°, and the characteristic peaks of PEO were located at 19.3° and 23.6°, based on the literature [43]. The characteristic diffraction peaks of chitosan were at 8.6°, 11.8°, 18.5°, and 23.1° [44,45]. The resulting PCL/PEO samples showed three characteristic peaks at 19.3°, 21.4°, and 23.6°, indicating that they contained both PCL and PEO components. The PCL/PEO/CS samples showed distinctive peaks for the PCL and PEO components, and more spurious peaks between 30° and 50°, which were the result of a chemical bonding reaction between PCL and PEO and CS. The addition of CS was not significant, so the characteristic peaks were not obvious. The peak positions of PCL/PEO/CS/CTAB_0.2_, PCL/PEO/CS/CTAB_0.4_, and PCL/PEO/CS/CTAB_0.6_ were consistent with each other, and only the peak intensities changed due to the different additions of CTAB, which led to different crystallinity of the prepared samples. The peak positions of CTAB were 6.6°, 16.9°, 20.4°, and 24.3° [46]. The XRD peak positions of PCL/PEO/CTAB_1_ were shifted to the left in comparison with the peaks of the three samples. There was a deviation in the thickness of the three materials and the sample was higher than the reference surface of the sample plate, resulting in the leftward shift of the diffraction peak [47], which was CTAB. In summary, the analysis tentatively concluded that the six composite films were prepared successfully.

**Figure 4:**
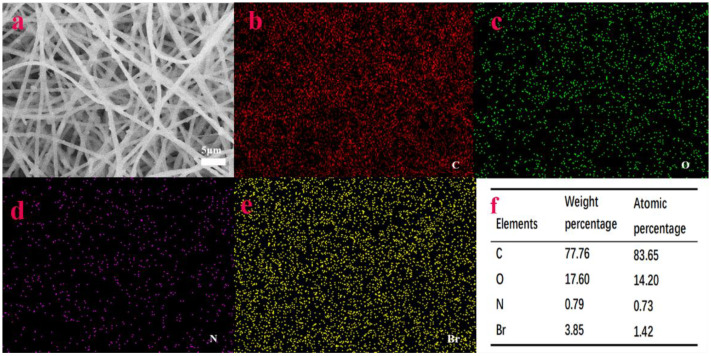
(**a**), Scanned images of the PCL/PEO/CS/CTAB_0.2_ composite structure; (**b**–**e**), elemental mapping of C/O/N/Br; (**f**), table of the elemental composition of C/O/N/Br.

Since the compositions of the three composite films PCL/PEO/CS/CTAB_0.2_, PCL/PEO/CS/CTAB_0.4_, and PCL/PEO/CS/CTAB_0.6_ were the same, only the ratios were different. FT-IR image analysis was performed for these four composite films to further analyze the internal composition of the samples. From Figure 3B, it can be seen that the spectral curves of a, b, c, and d were the same, and the locations where the absorption peaks appeared were the same, which could be identified as the characteristic absorption peaks of the PCL and PEO substrate polymers. Among them, 1725 cm^−1^ was the C=O stretching vibration peak [48], 2925 cm^−1^ and 2855 cm^−1^ were the methylene C-H stretching vibration peaks, and 1053 cm^−1^ to 1235 cm^−1^ were the C-O stretching vibration peaks, while 1350 cm^−1^ to 1462 cm^−1^ were the methylene C-H in-plane bending vibrations [47]. It can be seen that the molecular chains contained ester-based structures, as well as more hydroxyl units. This was the characteristic absorption peak of PCL polymers; while 845 cm^−1^ was the -CH2-CH2 stretching vibration peak, 1053 cm^−1^ to 1235 cm^−1^ was the C-O stretching vibration peak [49]. 1250 cm^−1^ was the -OH stretching vibration peak [50], 1725 cm^−1^ was the C=O stretching vibration peak, and 2855 cm^−1^ was the methylene C-H stretching vibration peak. These were the characteristic absorption peaks of PEO polymers. Because PCL and PEO components were present in each composite film, the characteristic absorption peaks of PCL and PEO appeared in all six composite films at these three locations. The small content of chitosan led to the overlap of the characteristic absorption peaks of the IR spectra with the other peaks, which can assist in inferring the presence of CS by the difference in antibacterial properties in the subsequent antibacterial experiments. In the IR spectra of PCL/PEO/CTAB_1_ and PCL/PEO/CS/CTAB_0.2_, no stretching vibration peak of C-Cl appeared. The relative atomic mass of halogenated hydrocarbons is very large, the infrared spectrometer is not stable in the detection of the stretching vibration of halogenated hydrocarbons, and the accuracy of the test results is low. Therefore, the successful doping of CTAB could not be proven in the IR spectrum, so the presence of elemental Br was detected using Mapping. The test results, shown in Figure 4, proved the presence of C, O, N, and Br elements in the structure of PCL/PEO/CS/CTAB_0.2_ and the uniform distribution of the elements, which proved the successful incorporation of the CTAB antimicrobial component.

**Figure 5:**
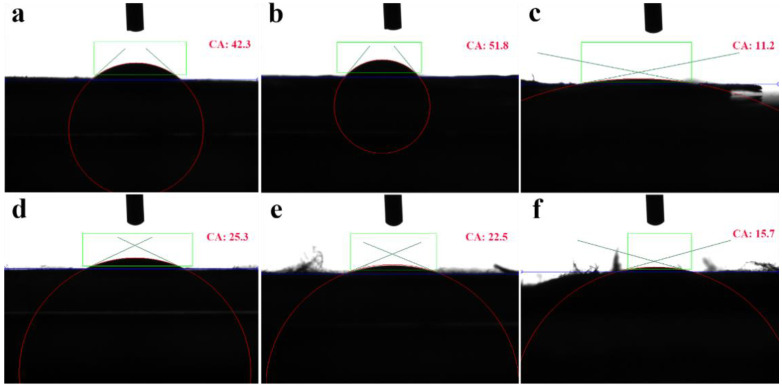
Water contact angle images of composite spinning films: (**a**), PCL/PEO; (**b**), PCL/PEO/CS; (**c**), PCL/PEO/CTAB_1_; (**d**), PCL/PEO/CS/CTAB_0.2_; (**e**), PCL/PEO/CS/CTAB_0.4_; (**f**), PCL/PEO/CS/CTAB_0.6_.

Materials with a water contact angle of less than 90° are considered hydrophilic, and those with contact angles greater than 90° are considered hydrophobic [51]. The hydrophilicity/hydrophobicity of the material influences to some extent the ease of antimicrobial agent release and the antimicrobial activity of the composite spinning film. Figure 5 shows the water contact angle of each composite film, and Figure 5a shows that the water contact angle of PCL/PEO was 42.253°. This was due to the presence of PEO in the composite spinning films, which is a strong hydrophilic material. Figure 5b shows the spinning film doped with CS based on PCL/PEO. Chitosan is insoluble in water, which enhanced the hydrophobicity of the spinning film to some extent, so the contact angle of the PCL/PEO/CS spinning film increased to 51.475°. The fiber in Figure 5c is the membrane based on PCL/PEO doped with a CTAB component, which has strong solubility water. The hydrophilicity of the fibers was obviously improved, and the contact angle was reduced to 11.222°. Figure 5d,e shows that with an increasing amount of hydrophilic CTAB, the water contact angles were 25.296°, 22.504°, and 15.679°, respectively. The hydrophilicity of the composite spinning film showed an increasing trend, which also provided favorable conditions for the release of the antimicrobial agent.

### 3.2. Antibacterial Performance and Analysis

**Figure 6:**
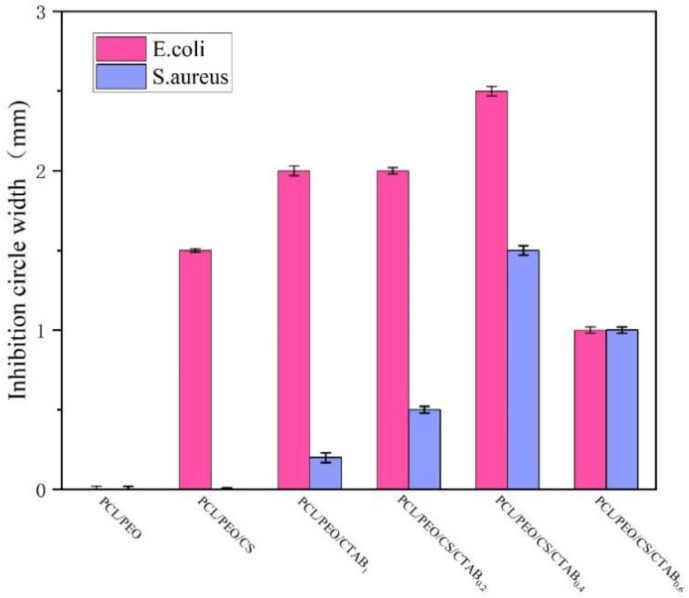
Inhibition circle width of six composite spinning films against *S. aureus.* and *E. coli*.

**Figure 7:**
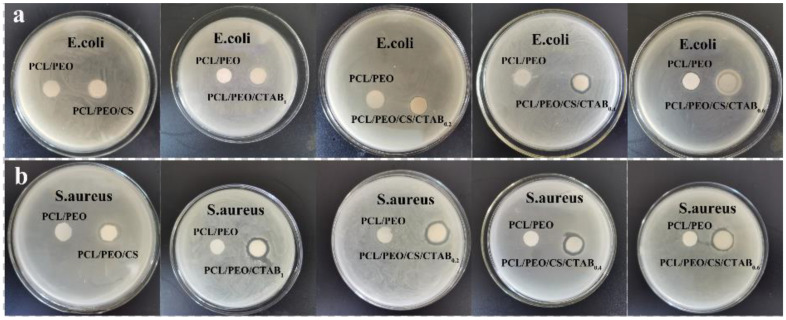
Images of the qualitative inhibition circle of composite films: (**a**) *E. coli*; (**b**) *S. aureus*.

It can be observed from Figure 6 and Figure 7 that PCL/PEO had no effect on Gram-positive and Gram-negative bacteria. This is because PCL/PEO does not contain antimicrobial agents that have an inhibitory effect on bacteria. Chitosan in PCL/PEO/CS has low antibacterial activity against *S. aureus*, with an inhibition zone width of 1 mm, and no effect on *E. coli*, which is due to the low chitosan addition and the fact that chitosan is an antimicrobial agent of natural origin that has a poor antibacterial effect. PCL/PEO/CTAB_1_ had a low antibacterial activity against *S. aureus*, with an inhibition zone width of 2 mm, and a zone of 0.2 mm against *E. coli*. The inhibition zone of PCL/PEO/CS/CTAB_0.2_ against *S. aureus* was 2 mm, while it was 0.2 mm against *E. coli*, while the inhibition zone of PCL/PEO/CS/CTAB_0.2_ against *S. aureus* was 2 mm, and it was 0.5 mm against *E. coli*. The inhibition effect of the composite films against *E. coli* was slightly enhanced by the addition of chitosan. The inhibition zone of PCL/PEO/CS/CTAB_0.4_ against *S. aureus* was slightly enhanced by the addition of chitosan. The width of the inhibition zone of PCL/PEO/CS/CTAB_0.6_ was 1 mm for *S. aureus* and 1 mm for *E. coli* when the amount of CTAB added was in a certain range. The contact area became larger, resulting in a reduction in the diameter of the inhibition circle.

In summary, PCL/PEO had no antibacterial effect against the tested bacteria, and the antibacterial effect of the remaining five composite spinning films varied, among which the antibacterial performance of PCL/PEO/CS/CTAB_0.4_ composite spinning films was the best, with an inhibition zone width of 2.5 mm for *S. aureus* and 1.5 mm for *E. coli*.

**Figure 8:**
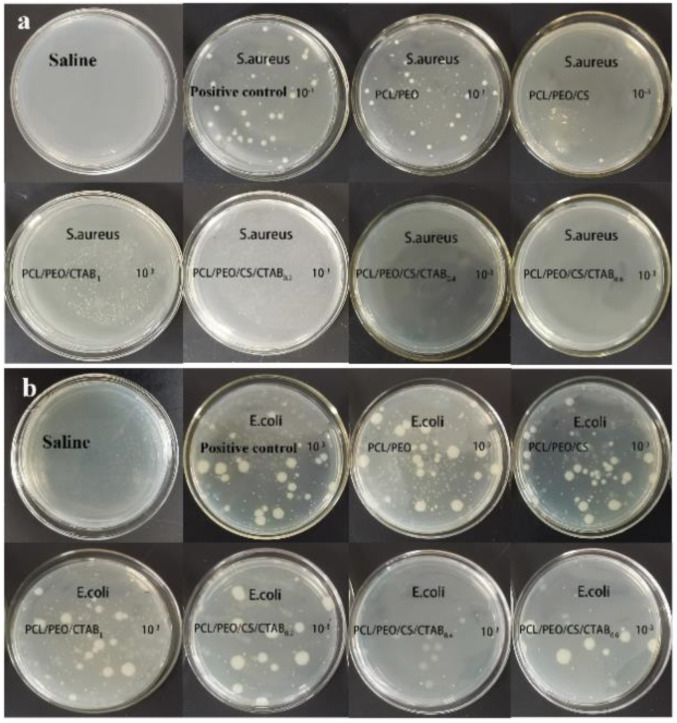
Images of the quantitative inhibition circles of composite films: (**a**) *E. coli*; (**b**) *S. aureus*.

**Figure 9:**
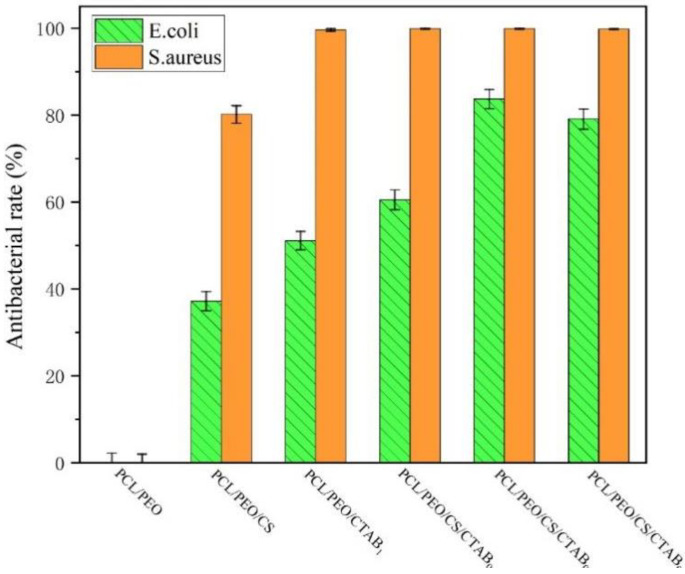
Images of the antibacterial rate of composite spinning films.

The antibacterial results of each composite spinning film against *S. aureus* and *E. coli* after treatment are shown in Figure 8 and Figure 9, from which it can be seen that, except for PCL/PEO composite films with no antibacterial activity, the rest of the composite films had a degree of antibacterial activity, and the antibacterial activity against the two strains was different. The antibacterial activity against Gram-positive bacteria (*S. aureus*) was higher, with the antibacterial rate was generally above 80%, while the antibacterial rate against Gram-negative bacteria (*E. coli*) was generally lower, which was also consistent with the results shown in the inhibition circle experiment. Although the peptidoglycan layer of Gram-positive cells is thicker than that of Gram-negative bacteria, the outer film of Gram-negative bacteria is surrounded by lipids and proteins, which can improve their resistance to the penetration of cationic antimicrobial polymers into the cells. On the other hand, the surface of Gram-positive bacteria has more negative charges, which makes them more resistant to the penetration of cationic antimicrobial polymers. It can be seen from the graph that PCL/PEO/CS/CTAB_0.4_ had the highest antibacterial rate of 83.7% for *S. aureus* and 99.9% for *E. coli*, which was due to the higher amount of antimicrobial agent in this composite film. However, the antimicrobial rate decreased. This was because there is a certain effective range for the amount of CTAB added, and when too much is added, the composite film becomes loose and the area of CTAB in contact with air becomes larger. Some is then lost, resulting in a decrease in the inhibition rate. This also corresponded to the experimental results of the inhibition circle.

**Figure 10:**
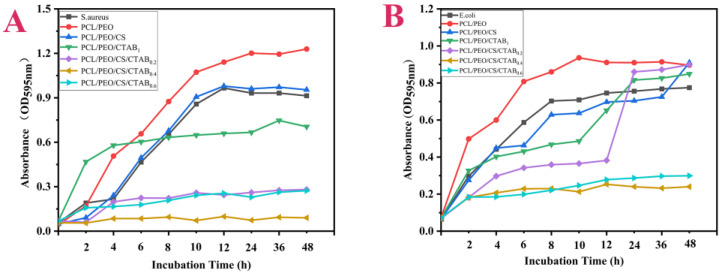
Inhibitory growth curves of composite spinning films: (**A**) *E. coli*; (**B**) *S. aureus*.

From Figure 10A, we can see that the absorbance of the bacterial growth curve of PCL/PEO was higher than the growth curve of pure *S. aureus*. Since this composite spinning film has a fiber structure that hides bacteria more easily and PCL/PEO does not have antimicrobial components and has no antimicrobial properties, the self-borne bacteria of the sample could not be completely eliminated by UV light irradiation alone, so when incubated in the shaker, the growth curves of PCL/PEO/CTAB_1_ and PCL/PEO/CS/CTAB_0.6_ were higher than the bacterial growth curves in the first eight hours. The macroscopic shapes prepared from these two samples were fluffy and easily harbored bacteria, so they were higher than the absorbance of bacteria, but the bacterial growth curves of the two samples entered the stabilization period earlier and exerted their antibacterial performance after 8 h and 2 h. The experimental results for PCL/PEO/CS, PCL/PEO/CS/CTAB_0.2,_ and PCL/PEO/CS/CTAB_0.4_, in the first two hours, were consistent with the experimental results of the inhibition circle and antibacterial rate, with good antibacterial activity. PCL/PEO/CS/CTAB_0.4_ showed the best and most stable antibacterial activity. As can be seen in Figure 10B, the logarithmic and stable regions of the bacterial growth curves of each composite spinning film, except PCL/PEO and PCL/PEO/CTAB, were below the pure bacterial control during the first two hours, indicating that these spun samples inhibited *E. coli*. 

PCL/PEO/CS had a consistent, weak, but stable inhibition of *E. coli*. The graph also showed that the degree of inhibition of the PCL/PEO/CTAB_1_ and PCL/PEO/CS/CTAB_0.2_ samples did not show a continuously increasing trend, but changed with the growth cycle of the bacteria. After 12 h, the bacterial absorbance of the two samples of PCL/PEO/CTAB_1_ and PCL/PEO/CS/CTAB_0.2_ increased significantly and the antibacterial rate decreased, which was due to the low number of bacteria in the first 0–12 h, the large concentration gradient of the antibacterial agent, and the rapid release rate of the antibacterial agent, so the antibacterial rate of the two composite spinning films increased rapidly. After 12 h, the bacteria entered the logarithmic growth curve stage, the reproduction rate rapidly increased, and the number of bacteria released from both samples increased, while the antibacterial dose re-released from both samples was lower than the reproduction rate of bacteria, resulting in a decrease in the inhibition rate. Regarding the release of Br ions to characterize the release of antimicrobial agents, we summarized the following points through the investigation of previous studies: First, from the analysis of drug release kinetics, the driving force of drug release is mainly the explanation process and diffusion process, and the degradation rate of the PCL/PEO structure used in this material is too slow [52], so the diffusion process is the main driving force, and the nano-level structure of this material facilitates the diffusion process. In addition, the pH growth range of *E. coli* is around 4.5–9, and the protonation of the amino group of CTAB is enhanced at lower pHs, resulting in an increasing positive charge and electrostatic repulsion in the polymer system, which leads to the release of the antimicrobial agent [53]. This makes the material suitable for use in lower pH environments.

The bacterial growth curve of PCL/PEO/CS/CTAB_0.4_ was more stable, and the absorbance was kept at a very low level, which indicated that the antimicrobial performance of PCL/PEO/CS/CTAB_0.4_ was the best among these samples and the antimicrobial performance was stable. This was also consistent with the inhibition circle and the antimicrobial rate results.

## 4. Conclusions

In summary, we successfully obtained PCL/PEO/CS/CTAB_x_ composite spinning films loaded with the natural antimicrobial material CS, together with the cationic antimicrobial agent CTAB, using an electrostatic spinning process. The blend of CTAB and CS enhanced the antimicrobial properties of the materials, and the prepared composite films were characterized by various methods. Water contact angle tests showed that the multiple materials of the vegetation were hydrophilic, which facilitated the release of the antimicrobial agent. In vitro antimicrobial activity studies demonstrated that PCL/PEO/CS/CTAB_0.4_ exhibited good and stable antimicrobial effects against both Gram-negative and -positive bacteria. These results suggested that the prepared composite spinning film materials can be used in applications such as masks and wound dressings to prevent infections.

## Data Availability

Data presented in this article are available on request from the corresponding author.
